# Biological Effects of Weak Electromagnetic Field on Healthy and Infected Lime (*Citrus aurantifolia*) Trees with Phytoplasma

**DOI:** 10.1100/2012/716929

**Published:** 2012-05-02

**Authors:** Fatemeh Abdollahi, Vahid Niknam, Faezeh Ghanati, Faribors Masroor, Seyyed Nasr Noorbakhsh

**Affiliations:** ^1^Department of Plant Sciences, School of Biology and Center of Excellence in Phylogeny of Living Organisms, College of Sciences, University of Tehran, Tehran 14155-6455, Iran; ^2^Department of Plant Science, Faculty of Biological Science, Tarbiat Modares University, Tehran 14115-154, Iran; ^3^Department of Chemistry, Engineering Research Institute, Sooliran Street, 16 km Tehran-Karaj Old Road, Tehran 13455-754, Iran

## Abstract

Exposure to electromagnetic fields (EMF) has become an issue of concern for a great many people and is an active area of research. Phytoplasmas, also known as mycoplasma-like organisms, are wall-less prokaryotes that are pathogens of many plant species throughout the world. Effects of electromagnetic fields on the changes of lipid peroxidation, content of H_2_O_2_, proline, protein, and carbohydrates were investigated in leaves of two-year-old trees of lime (*Citrus aurantifolia*) infected by the Candidatus *Phytoplasma aurantifoliae*. The healthy and infected plants were discontinuously exposed to a 10 KHz quadratic EMF with maximum power of 9 W for 5 days, each 5 h, at 25°C. Fresh and dry weight of leaves, content of MDA, proline, and protein increased in both healthy and infected plants under electromagnetic fields, compared with those of the control plants. Electromagnetic fields decreased hydrogen peroxide and carbohydrates content in both healthy and infected plants compared to those of the controls.

## 1. Introduction

During the past years considerable evidence has been accumulated with notice to the biological effects of low-frequency electromagnetic fields (EMF), such as those bringing from modern world such as power lines and household electrical wiring [1, 2]. The effects of electromagnetic fields of much lower frequency than visible light on plant growth and development have rarely been studied until relatively recently, and knowledge is still limited. Several studies have been showed that low-frequency EMFs may influence plant growth and development [3–5]. Also the international discussion about the biological effects of electromagnetic fields, in which we were involved in the past [6], led us to examine the possibility of using such fields to inhibit phytoplasmas growth on plants such as lime. Phytoplasmas are endocellular prokaryotes without cell wall associated with more than 600 diseases in at least 300 plant species [7]. Moreover, knowledge about phytoplasmas has been limited by inability to isolate them in pure culture.

Reactive oxygen substances (ROS), such as singlet oxygen, superoxide anion, and hydroxyl radical, are produced by a free radical chain reaction and may contribute to tissue damage. To mitigate the oxidative damage initiated by ROS, plants have developed a complex antioxidative defense system, including production of low-molecular mass antioxidants as well as antioxidative enzymes, such as superoxide dismutase (SOD), catalase (CAT), ascorbate peroxidase (APX), guaiacol peroxidase (GPX), and glutathione reductase (GR) [8]. Moreover, the level of malondialdehyde (MDA), a product of lipid peroxidation, has also been considered an indicator of oxidative damage under magnetic field [9].

Lime (family Rutaceae) is one of the most important and most economic horticulture products in the south part of Iran. Lime is susceptible to a large number of diseases caused by plant pathogens [10]. Witches' broom disease of lime (WBDL) associated with “*Candidatus *Phytoplasma aurantifolia” is one of the most destructive diseases of lime in the southern provinces of Iran [11]. Witches' broom disease of lime was first observed in the Sultanate of Oman and later was found to be present in United Arab Emirates [12], India [13], and Iran [14].

Studies on physiological relationships between phytoplasmas and some host plants have been reported [10, 15] but so far none have focused on the responses of phytoplasmas-infected lime plants to electromagnetic fields. Thus, the objective of the present work was to study some biochemical aspects related to lipid peroxidation, content of H_2_O_2_, proline, protein, and carbohydrates in phytoplasmas-infected lime plant under electromagnetic fields. 

## 2. Materials and Methods

### 2.1. Plant Materials and Electromagnetic Treatment

Lime plants (*Citrus aurantifolia* L. Swingle cv. Keyline) were infected with the Witches' broom disease of lime (WBDL) *Phytoplasma* by graft transmission and were grown in plastic pots (10 × 10 cm) under greenhouse condition in Engineering Research Institute, 16 km Tehran-Karaj Old Road, Iran. Infected lime plants used for grafting were collected from *Takht*, *Bandar-e-Abbas*, south of Iran and transported to the greenhouse. Shoots showing typical symptoms were grafted on two-years-old lime plants.

Exposure to EMF was performed by a locally designed electromagnetic wave generator able to generate different wave shapes including sinusoidal, triangular, and quadratic. The system could generate EMF in range of 0.1 Hz–10 KHz with a continuous fine control in stable conditions and maximum consuming power density of 9 W. It was consisted of two vertical coils each 28 turns of 0.3 mm copper wire rounded around a quadratic frame of 48 × 34 cm. One coil was oriented in vertical plane (XOZ) with pointing vector in horizontal direction (antiparallel with the gravity), while the other one was oriented in horizontal plane (XOY) with pointing vector in vertical direction (perpendicular with the gravity). Impedance of each coil was 8 ohm. The calibration of the system was performed at different frequencies. For the present experiment the healthy and infected plants were discontinuously exposed to a quadratic EMF for 5 days, each 5 h, at 25°C. The applied frequency was 10 KHz with consuming power of 8.3. The average electric and magnetic strength were  168.5 ± 4  (KV/m) and  3400 ± 43  (mA/m), respectively [16]. All measurements were conducted either in fresh harvested tissue ground immediately after excision or from leaves quickly deep frozen in liquid nitrogen and kept at −80°C until the assay.

### 2.2. Plant Water Relations

Leaf water content (WC) was calculated based on [17]

(1) $$\text{WC}\;\left(\%\right)=\left[\frac{\left(\text{fresh}\;\text{mass}-\text{dry}\;\text{mass}\right)}{\text{fresh}\;\text{mass}}\right]\times 100.$$

### 2.3. Protein Content

For determination of protein content, 500 mg fresh leaf was homogenized in a chilled (4°C) mortar using a 50 mM Tris-HCl buffer (pH 7.0) containing 10 mM EDTA, 2 mM MgSO_4_, 20 mM dithiotreitol, 10% (v/v) glycerol, and 2% (m/v) polyvinylpyrrolidone. After centrifugation at 13000 g for 45 min at 4°C, the supernatant was filtered and then transferred to Eppendorf tubes and the sample kept on ice at 4°C. A portion of eluent was stored at −70°C. Total protein content was measured by the spectrophotometric method of Bradford [18] using bovine serum albumin (BSA) as the standard.

### 2.4. Proline Content

Free proline content was determined according to Bates et al. [19] using L-proline as a standard. High-speed centrifuge (Beckman J2-21M, Palo Alto, USA) and UV-visible spectrophotometer (Shimadzu UV-160, Tokyo, Japan) with 10 mm matched quartz cells were used for centrifugation of the extracts and determination of the absorbance, respectively.

### 2.5. Malondialdehyde Content

The level of lipid peroxidation was measured in terms of thiobarbituric acid reactive substances (TBARS), following the method of Heath and Packer [20]. The plant materials (0.5 g) were homogenized in 5 mL of 0.1% (w/v) trichloroacetic acid (TCA) and centrifuged at 10,000 g for 20 min. To 1 mL aliquot of the supernatant, 4 mL of 0.5% thiobarbituric acid (TBA) in 20% TCA was added. The mixture was heated at 95°C for 30 min and quickly cooled in an ice bath. After centrifugation at 10,000 g for 15 min, the absorbance of the supernatant was recorded at 532 and 600 nm. The value for nonspecific absorption at 600 nm was subtracted. The concentration of MDA was calculated using extinction coefficient of 155 mM^−1^ cm^−1^.

### 2.6. Total Carbohydrates Content

For determination of carbohydrates content, 50 mg of dry powder was extracted using 10 mL of ethanol : distilled water (8 : 2; v/v), and supernatant was collected after twice centrifugation at 1480 g. The residue from ethanol extraction was subsequently used for polysaccharide extraction by boiling water [21]. Total carbohydrates content was estimated by the method of Dubois et al. [22].

### 2.7. Hydrogen Peroxide Content

The content of hydrogen peroxide was determined according to Sergiev et al. [23]. The plant materials (0.5 g) were homogenized in 5 mL of 0.1% (w/v) trichloroacetic acid (TCA) on ice and centrifuged at 12,000 g for 15 min. To 0.5 mL aliquot of the supernatant, 1 mL potassium phosphate buffer and 1 mL KI was added. The absorbance of the supernatant was recorded at 390 nm.

### 2.8. Statistical Analysis

All analyses were performed based on a completely randomized design. The data determined in triplicate were analyzed by analysis of variance (*ANOVA*) using *SPSS *(version *9.05*). Each data point was the mean of three replicates (*n* = 3). The significance of differences was determined according to Duncan's multiple range test (DMRT). *P* values < 0.05 are considered to be significant.

## 3. Result and Discussion

**Figure 1:**
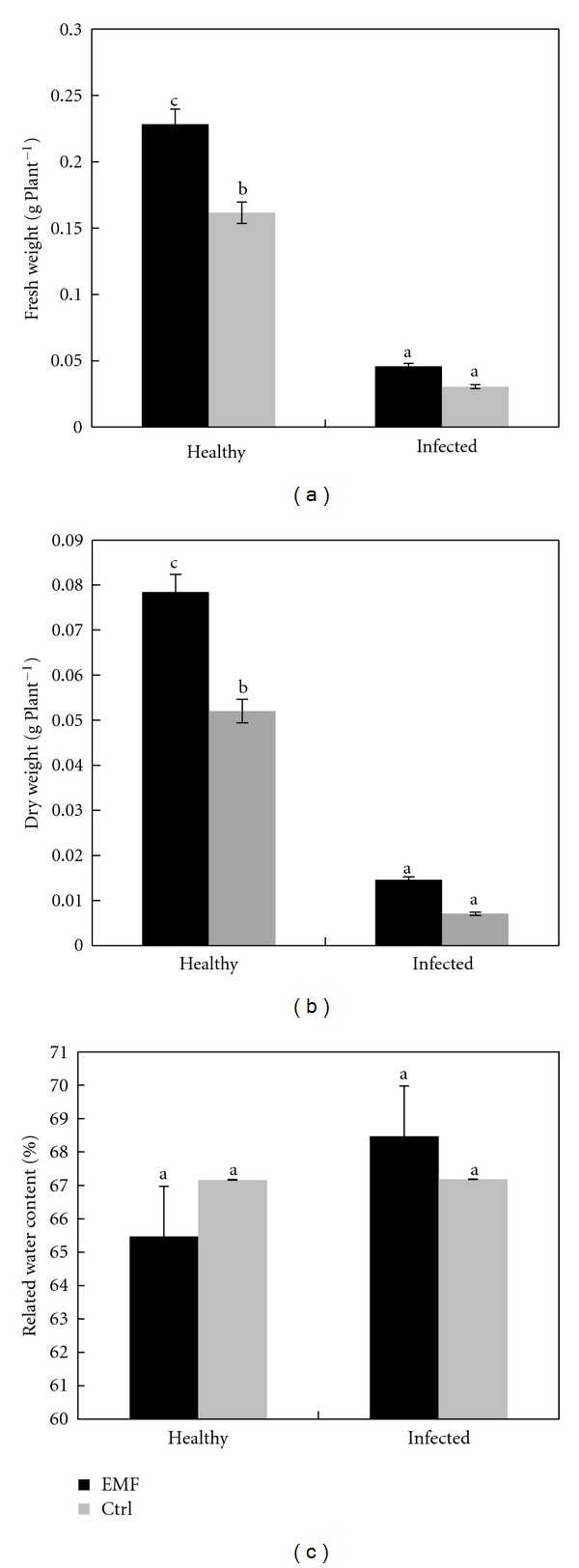
Effect of EMF on the fresh weight (a), dry weight (b), and relative water content (c) in healthy and infected plants of *Citrus aurantifolia.* Vertical bars indicate mean ± SE of three replicates. Different letters indicate significant differences (*P* < 0.05).

In order to determine the effect of electromagnetic field on leaf growth and biochemistry in *Citrus aurantifolia*, we treated the healthy and infected lime plants with electromagnetic field. The treatment of healthy and infected plants with electromagnetic field affected significantly the growth of the lime plants. Fresh and dry weight of leave in both healthy and infected plants increased under electromagnetic field (Figure 1). The present data agree with the previous results reported on *Prunus maritime*, *Cucumis sativus*, *Raphanus sativus*, and* Helianthus annuus *[24–26]. Relative water content (RWC) decreased in healthy plants and increased in infected plants under EMF stress (Figure 1(c)).

**Figure 2:**
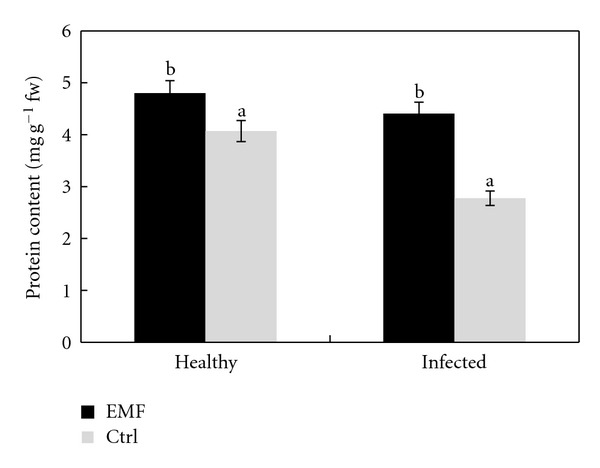
Effect of EMF on the protein content in healthy and infected plants of *Citrus aurantifolia.* Vertical bars indicate mean ± SE of three replicates. Different letters indicate significant differences (*P* < 0.05).

**Figure 3:**
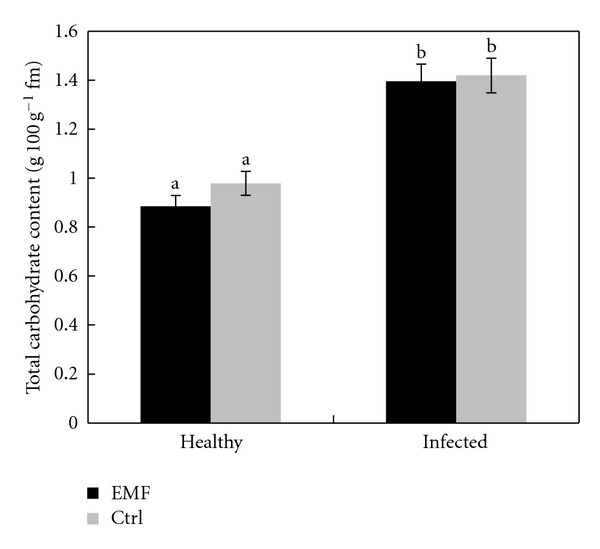
Effect of EMF on carbohydrates content in healthy and infected plants of *Citrus aurantifolia.* Vertical bars indicate mean ± SE of three replicates. Different letters indicate significant differences (*P* < 0.05).

Protein content in leaves of both healthy and infected plants increased significantly under EMF (Figure 2). EMF decreased slightly the content of total carbohydrates in both healthy and infected plants (Figure 3). The reduction in carbohydrates content is more prominent in healthy plants than that of infected ones.

**Figure 4:**
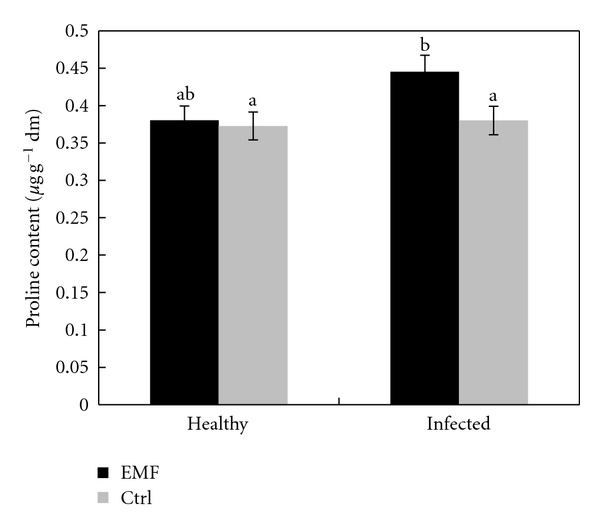
Effect of EMF on proline content in healthy and infected plants of *Citrus aurantifolia.* Vertical bars indicate mean ± SE of three replicates. Different letters indicate significant differences (*P* < 0.05).

Free proline content increased significantly under EMF exposure (Figure 4). However, enhanced levels of proline accumulation may not be enough to maintain water balance of the healthy plants under EMF treatment (Figure 1(a)). Many plants accumulated proline as nontoxic and protective osmolyte under stress conditions [27–29]. Higher accumulation of proline in lime plants under EMF may afford it much protection against electromagnetic field. Although the precise role of proline accumulation is still debated, proline is also considered to be involved in the preservation of cellular structures, enzymes, and to exploit as a free radical scavenger [30, 31].

**Figure 5:**
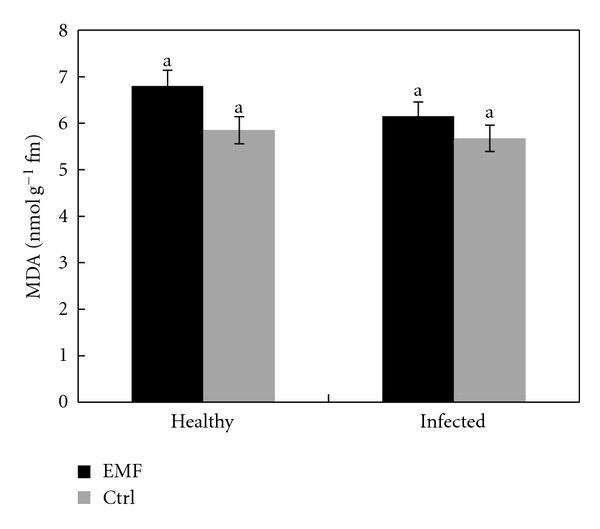
Effect of EMF on MDA content in healthy and infected plants of *Citrus aurantifolia.* Vertical bars indicate mean ± SE of three replicates. Different letters indicate significant differences (*P* < 0.05).

Changes in lipid peroxidation serve as an indicator of the extent of oxidative damage under stress, with an unchanged lipid peroxidation level seeming to be a characteristic of tolerant plants coping with elevated levels of stress. MDA content was higher in healthy and infected plants under EMF exposure (Figure 5). However these differences are not significant. Lipid peroxidation is mostly triggered by hydroxyl radicals, although the peroxidation can be caused by other reactive oxygen species as well. Treatment with the EMF did not cause any significant changes in the rate of peroxidation of the membrane lipids in lime plants. This result is against some results published on the detrimental effects of EMF [32, 33] and ELF-MF on membranes [9]. However, our result regarding MDA content is in accordance with the obtained result in maize under EMF [34] and rats under ELF magnetic field [35]. Damaging effects of magnetic field on DNA in animals are also reported [36].

**Figure 6:**
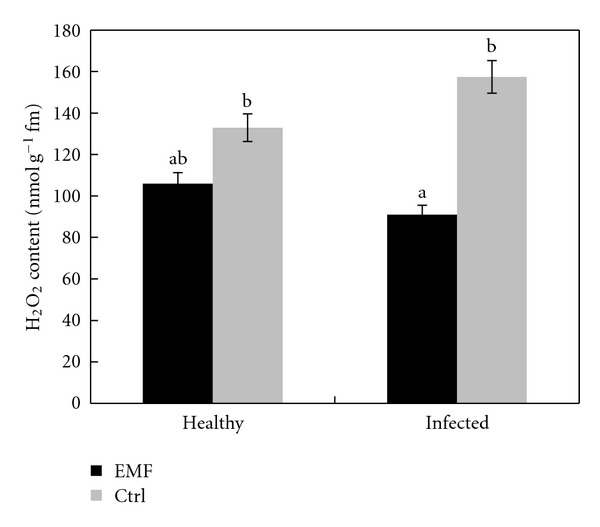
Effect of EMF on H_2_O_2_ content in healthy and infected plants of *Citrus aurantifolia.* Vertical bars indicate mean ± SE of three replicates. Different letters indicate significant differences (*P* < 0.05).

For determination of ROS scavenging capacity, the H_2_O_2_ content of lime plants under EMF stress was estimated. H_2_O_2_ content was always significantly lower under EMF stress throughout the experiments performed here (Figure 6). This result is in contrast to the obtained result on MDA content (Figure 5). Lower content of H_2_O_2_ might be a result of the significantly higher induction of the activities of antioxidant enzymes in the lime plants under EMF stress. Several authors have described the overproduction of toxic oxygen forms with aging in plants [37, 38]. It is known that ROS can cause peroxidation of membrane fatty acids [39]. In turn, these oxidized fatty acids may give rise to the propagation of peroxidation resulting in membrane damage. In a recent report, Lacan and Baccou [40, 41], besides supporting that ROS are involved in the ripening and senescence events in nonnetted muskmelon fruit, also showed that the observed delay in senescence in the long-storage life variety Clipper is closely linked to the very low free radical induced membrane lipid peroxidation in comparison with that of the short-storage life variety Jerac. These data explain the EMF-improved ability to scavenge free radicals, leading to a delay in senescence and alterations in membrane integrity, as demonstrated by the growth and survival responses. Furthermore, these results suggest that, under specific conditions, it takes the combined action of more than one antioxidant to provide an increased resistance to oxidative stress and lengthened survival in plants. This is in agreement with some previous reports [42, 43].

## 4. Conclusions

The obtained result show that 10 KHz EMF field can simulate the growth rate of healthy and infected lime plants. Moreover, it seems that EMF field could reduce the intensity of Witches' broom disease in infected trees and this effect could probably be due to the reduction of phytoplasma in plant tissues. This study provides an initial understanding of the response of infected lime plants to EMF stress, which is important for future studies aimed at developing strategies for struggle against phytoplasma and Witches' broom disease in lime plants.
